# Petrogenesis of juvenile pelletal lapilli in ultramafic lamprophyres

**DOI:** 10.1038/s41598-023-32535-2

**Published:** 2023-04-10

**Authors:** Ilya Prokopyev, Anna Doroshkevich, Anastasiya Starikova, Semen Kovalev, Yazgul Nugumanova, Andrey Izokh

**Affiliations:** 1 V.S. Sobolev Institute of Geology and Mineralogy Siberian Branch Russian Academy of Sciences, Akademika Koptyuga Avenue 3, 630090 Novosibirsk, Russia; 2 Novosibirsk State University, Pirogova Street 1, 630090 Novosibirsk, Russia; 3 Dobretsov Geological Institute, Siberian Branch of the Russian Academy of Sciences, Sakhyanovoy 6a, 670047 Ulan-Ude, Russia

**Keywords:** Solid Earth sciences, Petrology

## Abstract

The Chadobets alkaline-carbonatite complex (Siberian craton) is a natural laboratory for all varieties of ultramafic lamprophyres, including damtjernites formed by fluid-explosion mechanisms, which contain a large number of pelletal lapilli. Data obtained from comprehensive mineralogical, structural and chemical studies of these pelletal lapilli show strong similarity with the main magmatic mineral assemblage of damtjernites, suggesting a juvenile composition for them. The composition of phlogopite, carbonate and fluorapatite in pelletal lapilli is mostly constrained toward primitive compositions (beginning) of mineral crystallization trends in ultramafic lamprophyres. According to the petrographic and mineralogical features found in pelletal lapilli from damtjernites, these can be divided into three types based on the conditions and depth of formation. Estimated late magmatic temperatures of pelletal lapilli mica and fluorapatite formation vary from 815 to 990 °C.

## Introduction

Despite the relatively recent introduction into modern petrology of the ultramafic lamprophyres (UMLs) concept to refer to specific varieties of alkaline deep-mantle silicate-carbonate rocks such as aillikite and damtjernite^1,2^, currently, we are increasingly detecting the occurrence of these rocks among carbonatitic complexes, kimberlites and related rocks. Researches pay special attention to the genesis and evolution of specific alkali silicate-carbonate melts^2–17^. A fundamental question that yet remains open is petrogenetic, and has to do with how such great diversity of alkaline rocks is linked to one another. Petrological investigations have shown, however, that UMLs are generated by melting of carbonate-rich peridotite mixed with phlogopite-bearing metasomes^2–17^. The formation of alkali-ultramafic-carbonatite complexes depends on several controlling factors such as: composition of the primary melt, degree of partial melting, assimilation of crustal materials, mechanisms of melt separation and crystallization (i.e., immiscibility and/or fractional crystallization), speed and transportation mechanism of the melt to the surface, as well as other magmatic processes occurring in intermediate magma chambers.

The term “pelletal lapilli” is not new to petrology and volcanology but the mechanisms concerning their origins remain controversial^18–22^. Pelletal lapilli have been described in alkaline-carbonatite, melilite, and predominantly in kimberlite systems, and are represented by spherical formations with a central seed fragment, to which material of juvenile origin sticks and rapidly crystallizes, brought to the surface as a result of explosive eruptions of ultramafic magmas^18–24^. ‘Cognate inclusion’ and ‘autolith’ have also been used to describe these features^2^. The latest evidence shows that pelletal lapilli are formed when fluid melts intrude earlier volcanic formations, and intensive degassing produces a gas jet in which locally scavenged particles are simultaneously fluidized and coated by droplets of low-viscosity melt^25^. In any case, pelletal lapilli are key in providing information about the composition and evolution processes of the parental fluidized alkaline silicate-carbonate melt. Therefore, extensive studies of such “globular segregations” are desirable and recommended.

The Chadobets alkaline-carbonatite complex (Siberian craton) is known for its typical examples of various ultramafic lamprophyres, including damtjernites formed by fluid-explosion, which contain a large number of pelletal lapilli. A vast REE-Nb deposit, in terms of reserves, named Chuktukon, accumulated in the weathering crust of carbonatites, is also found within this complex. Accordingly, the petrological data in this study significantly contribute to both the fundamentals of alkaline magmatism and the discovery of strategic metal reserves. It is worth noting that this detailed investigation on pelletal lapilli of ultramafic lamprophyres is the first of its kind, but the occurrence of such features had been noticed at the type locality of carbonate-rich UMLs in Labrador, Canada^2^.

## Geology

**Figure 1:**
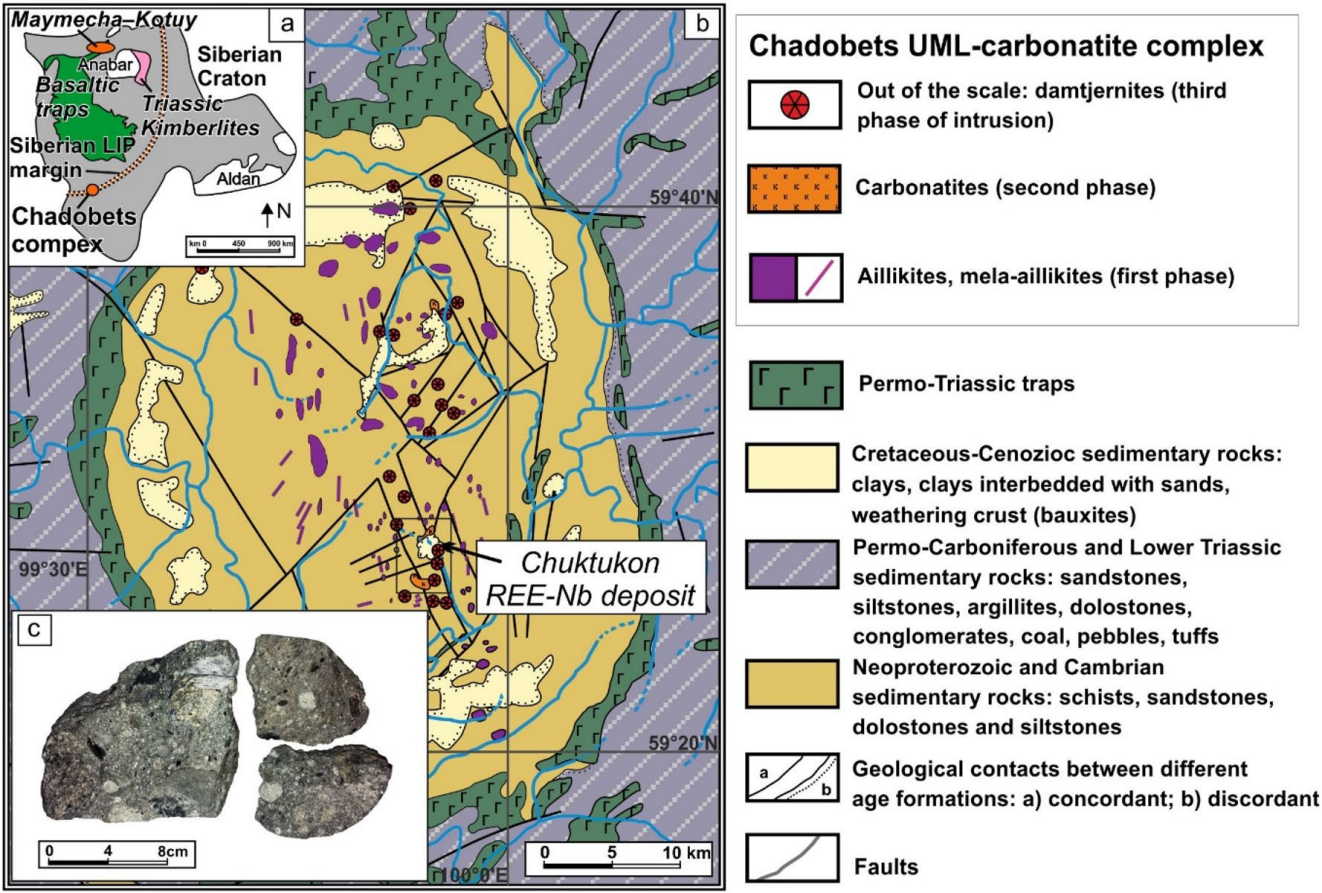
(**a**) Location of the Chadobets ultramafic lamprophyre (UML)–carbonatite complex within the Siberian Large Igneous Provence (LIP)^27^. (**b**) Geological scheme of the Chadobets alkaline complex^7,14^. (**c**) Samples of damtjernites from exploratory well (548–100.8 m). The figure was created using Corel Draw X4 software https://www.coreldraw.com/en/pages/coreldraw-x4/.

The Chadobets UML-carbonatite complex is located on the southwestern part of the Siberian craton, and within the southernmost margin of the Siberian superplume (Fig. 1a). The exploration history of this area is primarily associated with regional geological surveying and prospecting for diamonds, bauxites, as well as REE-Nb ores of the Chuktukon deposit (Fig. 1b). Crystallization age of rocks from the Chadobets complex lies within the range of 255–240 Ma^7,8,11,26^, which coincides with the Paleozoic–Mesozoic magmatic events of the Siberian large igneous province (LIP). The emplacement of the Chadobets alkaline rocks was coeval with intrusions associated with the flood basalts^27,28^, carbonatites and UMLs of the Maymecha–Kotuy province^29–32^, as well as with Siberian kimberlites from Anabar and Olenek region, and some lamproite intrusions^27,33–35^ (Fig. 1a).

Geological and textural data indicate that several pulses of alkaline magma formed the Chadobets UML-carbonatite complex. The aillikites and mela-aillikites comprise the first stage of the alkali-rich emplacement (Fig. 1b). These rocks form dyke swarms and sills ranging in length from 1–3 up to 120 m. The ore-bearing carbonatites crystallized at a second stage forming intrusive bodies such as stocks (up to 2.5 × 1.5 km), dykes, and sills of 20 m width by 2–3 km in length. The last magmatic phase within the complex is represented by damtjernite dykes and pipes usually 150 × 200 m in size near the surface (Fig. 1b)^7,8,11,12,14,26^.

The Chadobets UML–carbonatite complex is confined to the Chadobets plateau, which extends N–S forming an elongated structure of roughly 40 × 50 km (Fig. 1b). The origin of the Chadobets plateau is linked to an ascending diapir, of which geophysical data show the presence of an intermediate magma chamber at about 4–8 km depth^7,14^.

Cretaceous–Cenozoic sediments mainly composed of clay and clays interbedded with sands including a weathering crust forming bauxites cover intrusions of the Chadobets complex (Fig. 1b). The rock formations surrounding the Chadobets alkaline complex correspond to Neoproterozoic and Cambrian sedimentary packages such as shales, sandstones, dolostones, and siltstones. The periphery of the Chadobets highland comprises Permian-Carboniferous and Lower Triassic sediments: sandstones, siltstones, argillites, dolostones, conglomerates, coal, pebbles, tuffs, and areas of Permo-Triassic basalts (Fig. 1b).

The damtjernite pipes cut earlier alkaline rocks and usually contain their xenoliths as well as country rock fragments (Fig. 1c). In general, all types of magmatic rocks of the Chadobets complex were subjected to intense hydrothermal-metasomatic alteration and weathering processes. The pelletal lapilli occur in abundance within explosive pipes of damtjernites, fresh samples of which were obtained during exploratory drilling (Fig. 1c). Petrological and mineralogical features of the alkaline rocks in the Chadobets complex have been previously described^7,8,10–12,14,16,36^. Below we provide new details on the mineral compositions of damtjernites as well as the origin and composition of pelletal lapilli from the Chadobets UMLs.

## Results

### Mineralogy of damtjernites and pelletal lapilli

**Figure 2:**
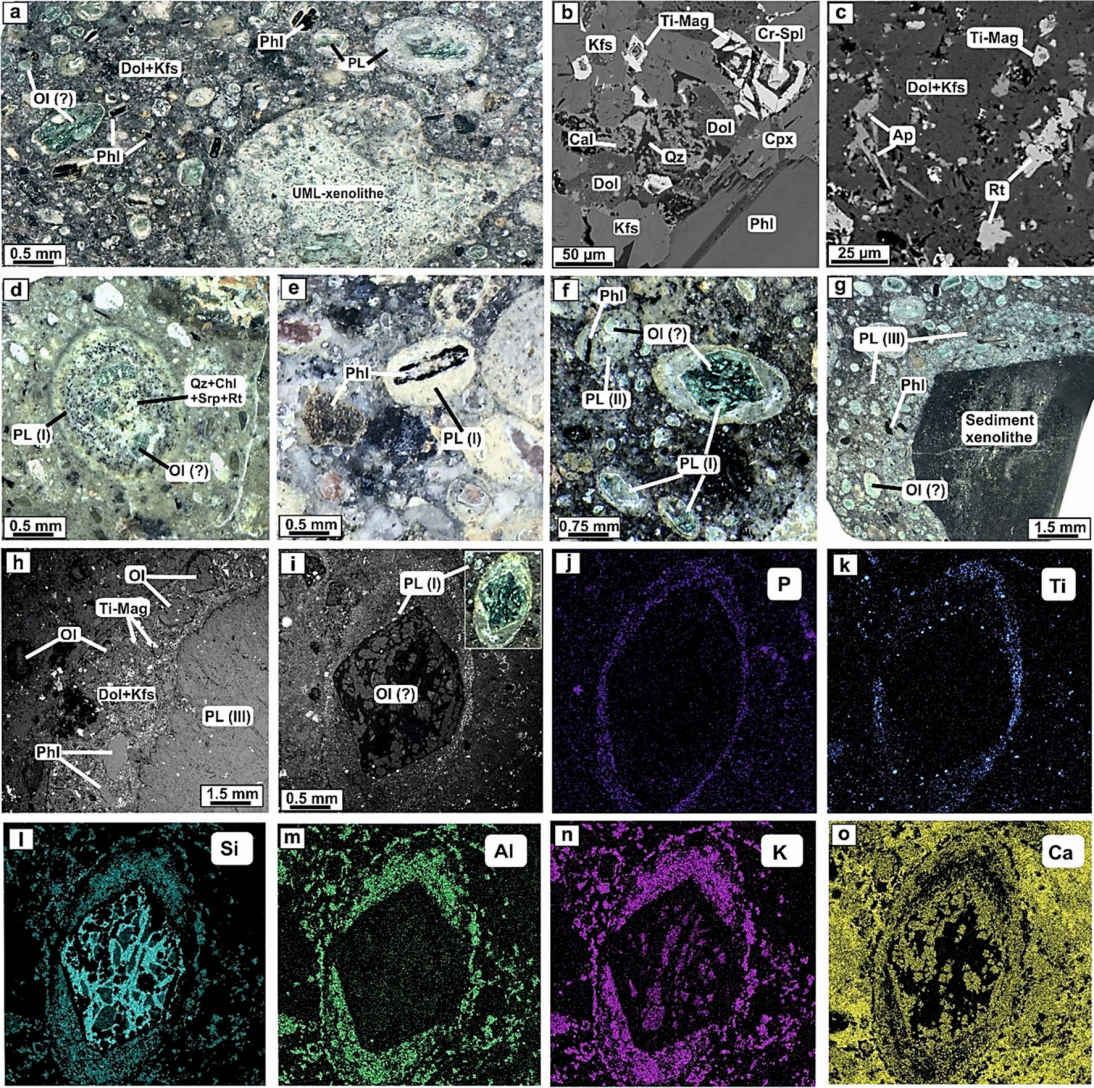
Photomicrographs (**a,d–g**) and back-scattered electron (BSE) images (**b,c,h,i**) of mineral assemblages of the Chadobets damtjernites with xenoliths of the earlier formed UML rocks (UML-xenolithe) and pelletal lapilli (PL) inclusions (**a**). Mineral composition of the damtjernites includes macrocrysts and phenocrysts of phlogopite (Phl) and olivine (Ol) within a groundmass of microphenocrysts of phlogopite, K-feldspar (Kfs) and dolomite (Dol), predominantly comprising clinopyroxene (Cpx), fluorapatite (Ap), Cr-spinel (Cr-Spl), Ti-magnetite (Ti-Mag) and rutile (Rt) crystals and grains (**b,c**). Pelletal lapilli type I (PL-I) contains olivine (Ol?) or phlogopite grains in in the cores, which are often completely replaced by quartz (Qz)-chlorite (Chl)-serpentine (Srp) with calcite (Cal) and rutile aggregates (**d–f**). The pelletal lapilli of the second type (PL-II) comprise two and or more minerals of phlogopite and olivine (Ol?) in the central area (**f**). The center of pelletal lapilli type III (PL-III) consists of lithic fragments of country rock or earlier alkaline pulses (xenoliths), whereas the rim of is represented by the damtjernites mineral assemblages (**g,h**). The mantle composition of PL-type I is shown on SEM elemental maps and includes P, Ti, Si, Al, K and Ca (**i–o**).

Damtjernite pipes of the Chadobets UML-carbonatite complex exhibit a porphyritic structure consisting of macrocrysts and phenocrysts of olivine and phlogopite (20–50 vol.%) within a groundmass of predominantly phlogopite, K-feldspar and dolomite composition with minor clinopyroxene, fluorapatite, Cr-spinel, Ti-magnetite, and ilmenite crystals and grains (Fig. 2a–c). Damtjernites often contain xenoliths of the earlier formed UML rocks and carbonatites, fragments of sedimentary rocks, as well as abundant pelletal lapilli inclusions (Fig. 2a–g). Secondary minerals in the damtjernites include quartz, calcite, serpentine, epidote, chlorite and rutile, replacing the magmatic minerals (Fig. 2b–d).

Pelletal lapilli found in these damtjernites can be divided into three types according to the mineral composition of the core (seed minerals or rock fragments) (Fig. 2d–i). The first type (PL-I) is formed around large single macrocryst seeds (and/or phenocrysts) of olivine (?) or phlogopite; the core minerals are usually 1–8 mm in size (Fig. 2a,d–f). The second type pelletal lapilli (PL-II) contains several crystal seeds of relatively smaller size (100–500 µm) "in intergrowth" with juvenile mineral assemblage (Fig. 2f). The third type pelletal lapilli (PL-III) corresponds to fragments of country rocks or earlier magmatic pulses of the Chadobets complex (Fig. 2g,h). In some cases, the size of the seed fragments can reach up to several cm, while the dimension of the mineral phases on the marginal sections of the lapilli is within an order of magnitude greater than those phases on the rim in the pelletal lapilli-I and the mineral assemblages of pelletal lapilli-II (Fig. 2g,h).

The mineral composition on the marginal zones (rims) of the pelletal lapilli among all three types is quite similar and represented by mineral assemblages comparable to those making up damtjernites. However, it should be noted that both the mineral phases of the seed kernels, as well as the mantle zones of the pelletal lapilli underwent intensive hydrothermal-metasomatic alteration (silicification, chloritization, serpentinization, carbonatization, etc.), which significantly obscures their primary (magmatic) chemical composition (Fig. 2d–f). Nonetheless, different types of pelletal lapilli show some chemical heterogeneity in the composition of marginal phases in pelletal lapilli, which may be clearly observed on the elemental maps obtained by SEM-EPMA and Raman spectroscopy. The combination of these two methods made possible to obtain better characterization of the mineral composition and distribution patterns of mineral phases within pelletal lapilli.

**Figure 3:**
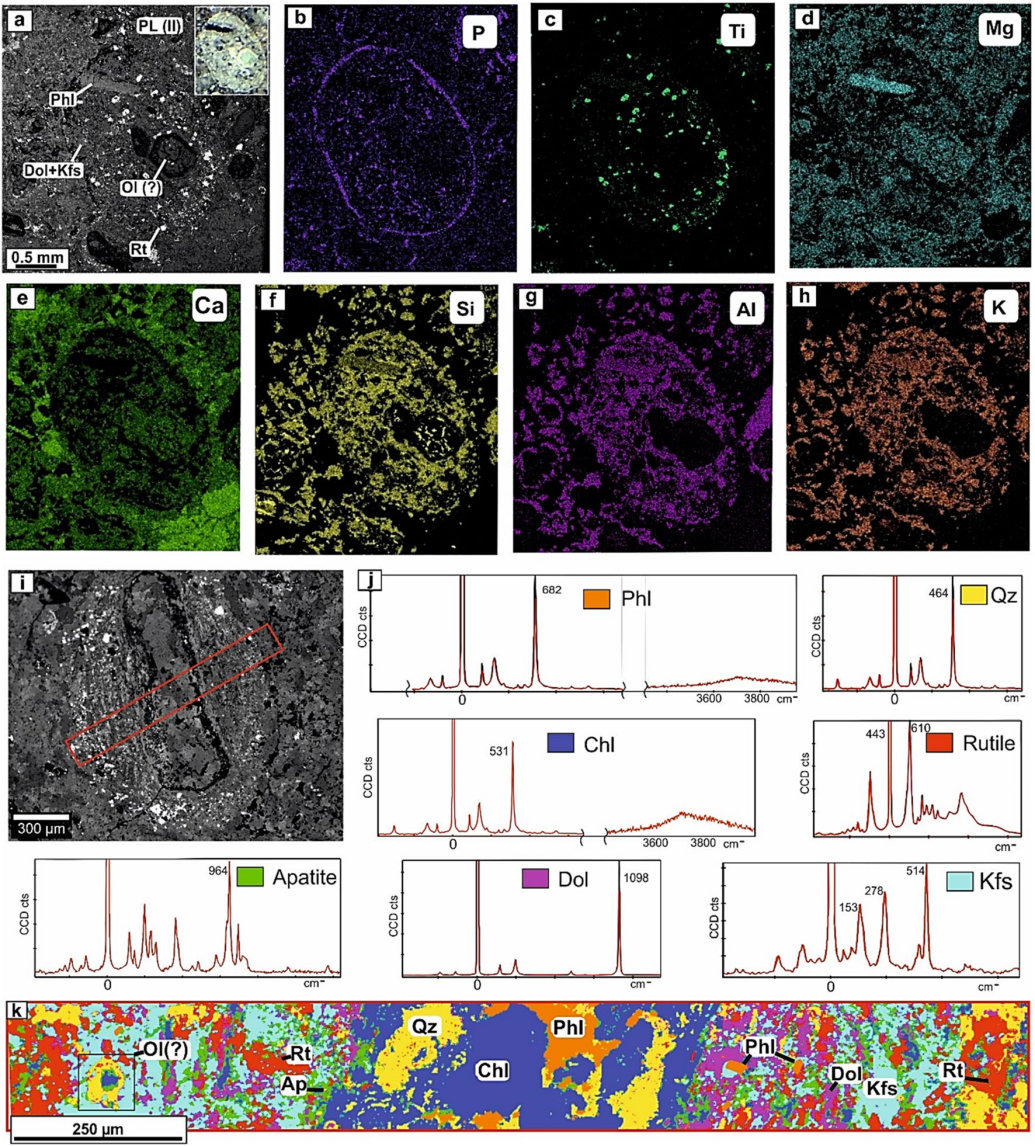
BSE image with a photomicrograph inset of the type II pelletal lapilli (PL-II) (**a**) and the rim composition of the pelletal lapilli-II as observed in SEM elemental mapping with distribution of P, Ti, Mg, Ca, Si, Al and K (**b–h**). Results from the Raman study of type I pelletal lapilli (PL-I) sample: BSE image of PL-I (**i**): Raman spectra of identified crystal phases (**j**) and mineral distribution map of PL-I (**k**) obtained by areal Raman mapping on the highlighted red section in the BSE image (**i**). The figure was created using Corel Draw X4 software https://www.coreldraw.com/en/pages/coreldraw-x4/.

Pelletal lapilli type I and II are markedly zoned in terms of their chemical composition (Figs. 2 and 3), being pelletal lapilli-I rhythmically zoned to the margin, where several compositional bands (usually 2–3) of P, Ti, Al, K, Ca and Si (Fig. 2i–o) may be observed. On the other hand, such rhythmic zoning pattern is not observed for the pelletal lapilli-II, although a clearly developed zoning is evident (Fig. 3).

The composition as determined by Raman spectroscopy showed the structural presence and distribution of different mineral phases (Fig. 3). Raman spectra of the first two types of pelletal lapilli (PL-I and PL-II) confirmed the presence of phlogopite grains as kernels and on the margins of the pelletal lapilli; the presence of rutile and fluorapatite grains zonally distributed in a matrix of K-feldspar-dolomite; in addition, a few relics after olivine are found on the rim structures. A profound alteration of primary magmatic minerals in the pelletal lapilli by quartz-chlorite aggregates is also observed (Fig. 3).

**Figure 4:**
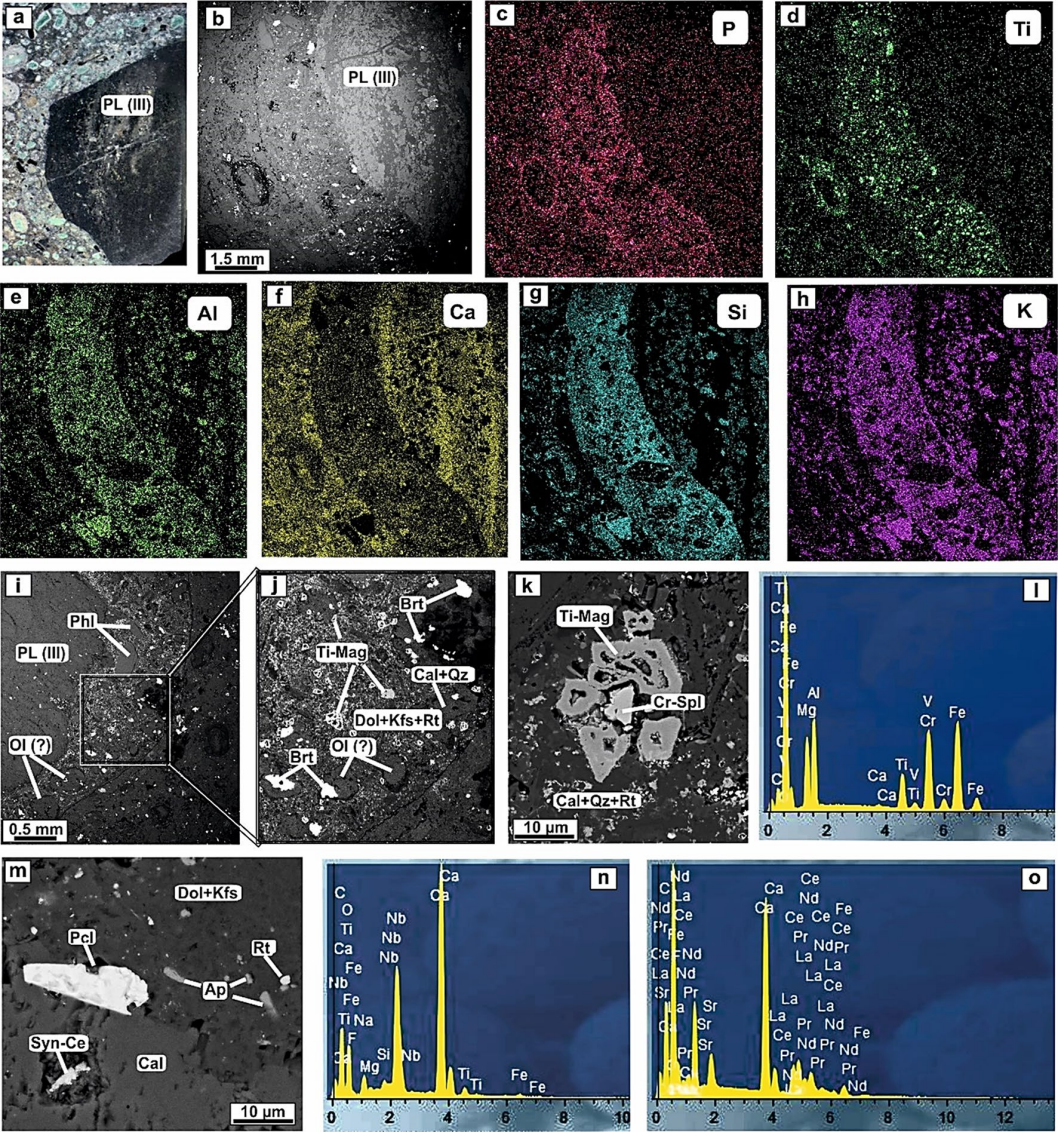
Photomicrograph (**a**) and BSE image of type III pelletal lapilli (PL-III) (**b**), as well as the composition of its rim according to SEM elemental mapping showing the distribution of P, Ti, Al, Ca, Si and K (**c-h**). BSE images (**i–k,m**) and SEM-WDS spectra of minerals: Cr-spinel (Cr-Spl) (**l**), pyrochlore (Pcl) (**n**) and synchysite-(Ce) (Syn-Ce) (**o**) making up the rim mineral assemblage of pelletal lapilli-III. Other phases routinely identified include olivine (?), phlogopite, apatite and Cr-spinel-Ti-magnetite primary grains located in a K-feldspar-dolomite matrix with secondary calcite, quartz, rutile, barite and synchysite-(Ce).

Meanwhile, the juvenile rim of type III pelletal lapilli does not have a marked zoning (Fig. 4a–h). The mineral composition of the rim in pelletal lapilli-III is consistent with that of the first two types (I and II) and represented by phlogopite and olivine (?), fluorapatite, rutile (Nb), titanomagnetite, spinels (Cr), barite as well as rare phases such as pyrochlore and rare earth minerals—monazite-(Ce) and synchysite-(Ce),—mantled by a matrix rim of K-feldspar-dolomite composition (Fig. 4i–o). Quartz, chlorite, calcite, epidote and some iron oxides and hydroxides represent a secondary mineralization.

*Olivine (Ol?)* phenocrysts as well as crystal kernels in all types of pelletal lapilli are commonly replaced by assemblages of secondary minerals such as quartz, chlorite, calcite, and serpentine with rutile impregnations. (Fig. 2d–f,i; Fig. 3, Fig. 4) where only relics are left. All macrocrysts are completely altered.

**Figure 5:**
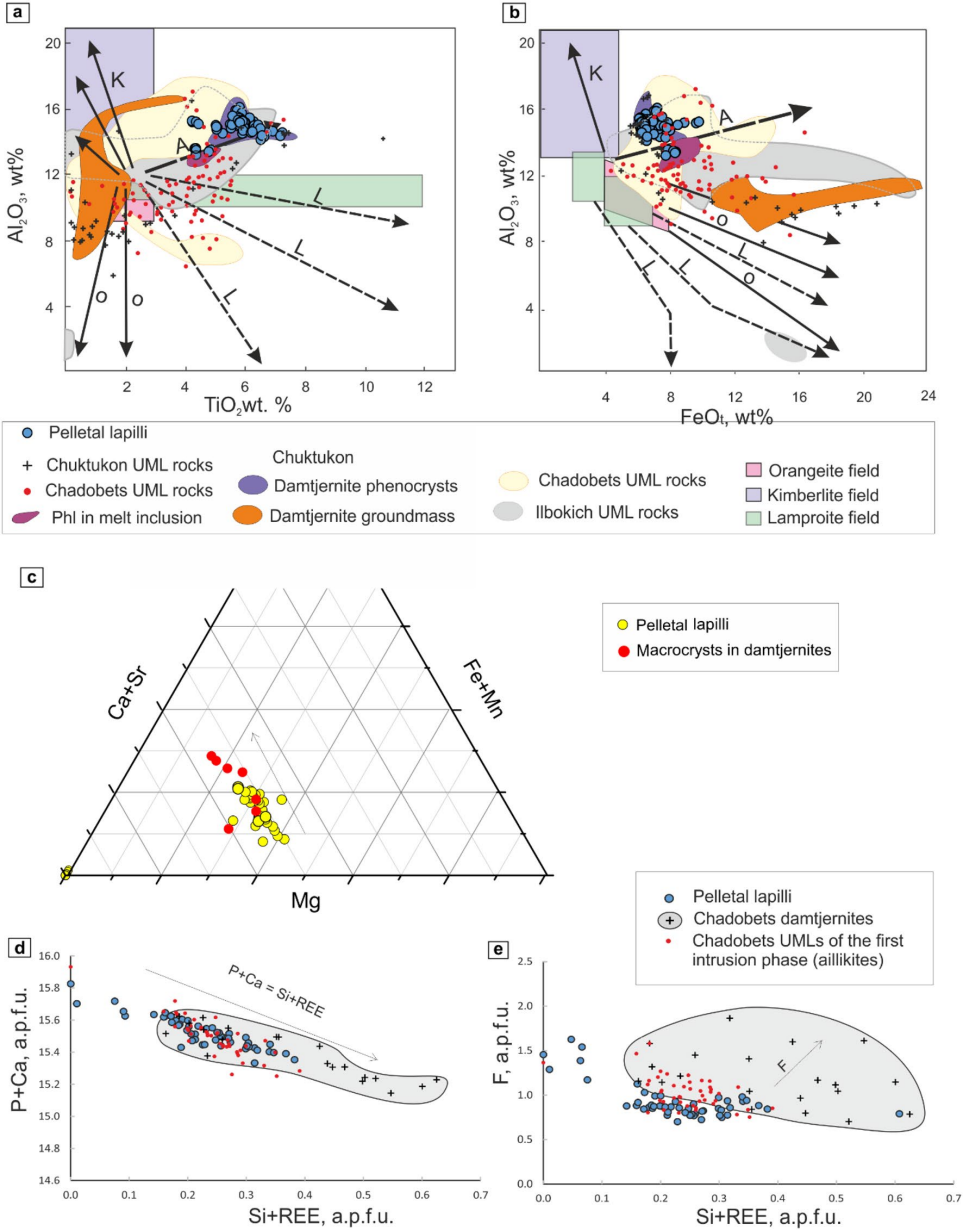
Compositional trends of phlogopites from the Chadobets complexes^7,11,14^. Al_2_O_3_ vs. FeOt (**a**) and TiO_2_ diagrams (**b**). UML rocks: K—kimberlite trends, O—orangeite trends, L—lamprophyre trends, M—minette trend^19^. Compositional evolution of carbonates from the Chadobets complex in a ternary diagram of Ca + Sr vs. Mg vs. Fe + Mn (**c**). The composition of fluorapatite in pelletal lapilli develops a compositional trend with carbonates of damtjernites^7^; whereas secondary calcite diverges from this trend. Compositional trends of fluorapatites from pelletal lapilli and damtjernites and aillikites of the Chadobets complex; considering the a.p.f.u variations between P + Ca vs. Si + REE (**d**) and F vs. Si + REE (**e**).

*Phlogopite* crystals in all three types of pelletal lapilli were investigated using an electron microprobe analyzer (Table S1). The chemical composition of mineral phases among them is similar and varies: 4.22–6.56 wt% of TiO_2_, up to 0.79 wt% of BaO, up to 0.06 wt.% of MnO (%), and 0.17–1.83 wt% of Cr_2_O_3_ (Table S1). The phlogopite compositional range in pelletal lapilli is similar to that of phenocrysts in damtjernites of the Chadobets complex, which indicates a juvenile mineral composition in pelletal lapilli (Fig. 5a,b). Moreover, this phlogopite mineral composition lies within the compositional fields of mica from the Chadobets and Ilbokich UML complexes, and which belong to the same geological structure^7,11,14,19^ (Fig. 5a,b).

Minerals of the *spinel group in* damtjernites as well as in UMLs of the Chadobets complex occur as composite subhedral zoned crystals. The central parts exhibit Cr-spinel compositions with *Ti-magnetite* rims (Fig. 2b,c). A similar distribution in the composition is observed for spinels in pelletal lapilli (Fig. 4k). The skeletal Ti-magnetite grains are 10–20 µm in size and consist of up to 1.84 wt% MgO, 1.02–3.66 wt% Al_2_O_3_, 11.46–13.5 wt% TiO_2_, up to 0.84 wt% MnO, and 75.32–81.49 wt% FeO_t_. Meanwhile, smaller spinel crystals about 5–15 µm in size, contains 1.41–30.99 wt% Cr_2_O_3_, 44.05–67.43 wt% FeO_t_, 4.91–9.56 wt% Al_2_O_3_, and 6.02–16.88 wt% TiO_2_ comparable to the composition of spinel group minerals from UMLs of the Chadobets complex^7,14,36^.

The dominant carbonate phase in damtjernite pelletal lapilli is *dolomite*, whereas *calcite* is subordinate (Fig. 4m; Table S2). Carbonates occur as anhedral grains about 15–25 µm in size. The dolomite contains 10.95–15.64 wt% MgO, 27.38–28.99 wt% CaO, 5.66–12.04 wt% FeO_t_, 0.86–2.21 wt% MnO, 0.12–0.52 wt% SrO, up to 0.08 wt% Nd_2_O_3_ and up to 0.06 wt% Ce_2_O_3_ (Table S2). The composition of secondary calcite is characterized by a significant depletion in concentrations (wt%) of MnO (0.07–047), FeO_t_ (0.12–0.4) and SrO (up to 0.18) (Fig. 5c), as well as the LRRE_2_O_3_, which is below detection limit (Table S2). The dolomite composition in pelletal lapilli lies at the beginning of the evolutionary trend of carbonates from the Chadobets damtjernites (Fig. 5c)^7^.

The dolomite is intergrown with *K-feldspar* in the pelletal lapilli matrix, similarly to the matrix of damtjernites (Figs. 2b,c, 3a, 4j,m). K-feldspar occurs as anhedral grains (5–15 µm); and it is mostly orthoclase (Or_77_–_100_) with Na_2_O up to 0.84 wt%. The BaO is below the detection limit. The composition of K-feldspar in pelletal lapilli is analogous to that of the damtjernite from the Chadobets complex^14^.

*Fluorapatite* appears forming euhedral grains and prismatic crystals of 5–25 µm in size, considered the less abundant primary mineral in damtjernites and pelletal lapilli, and formed at a late magmatic stage (Figs. 2c and 4m). The fluorapatite in pelletal lapilli contains (wt%): up to 1.87 SiO_2_, 0.05–1.09 Na_2_O, 0.84–1.48 SrO, up to 1.63 LREE_2_O_3_, and up to 0.11 ThO_2_ (Table S3). The composition of fluorapatites in pelletal lapilli shows an evolution trend, where Si and REE notably increase replacing isomorphically the position of P and Ca (Fig. 5d). In addition, we plotted the compositions of fluorapatites from UMLs of the early intrusion phase of the Chadobets complex, as well as the fluorapatites from the damtjernites groundmass (Fig. 5d–e; Table S4). A close similarity in the composition of fluorapatites from pelletal lapilli and the early stage lamprophyres, as well as the location pelletal lapilli fluorapatite at the trend beginning is clearly observed (Fig. 5d,e), thus, indicating a juvenile composition of fluorapatite in pelletal lapilli in relation to the magmatic evolution of the alkaline complex. A steady increase in the fluorine content is shown for the composition of damtjernite fluorapatites, which evidences that fluorapatites with lower fluorine correspond to earlier mineral phases (Fig. 5e).

Meanwhile, quartz, calcite, serpentine, chlorite, rutile, pyrochlore, barite, synchysite-(Ce), and monazite-(Ce) represent secondary minerals in pelletal lapilli (Figs. 2, 3i and 4i–o). These mineral association is thought to be related to the hydrothermal-metasomatic alteration stage of damtjernites^7,14^ and pelletal lapilli, which replaces magmatic minerals and form diverse networks of microveinlets and micrograin aggregates (microlites).

Nb-containing minerals are represented by rutile and pyrochlore. *Rutile* replaces Ti-magnetite and iron-bearing minerals (e.g., phlogopite), forming spots in pseudomorphs minerals after olivine, as well as microgranular aggregates within the pelletal lapilli K-feldspar-dolomite and calcite matrix. Rutile contains 0.56–2.35 wt% Nb_2_O_5_.

*Pyrochlore* is rare and occurs as euhedral grains with size of about 5–25 µm. It is often associated with apatite and rutile in the pelletal lapilli K-feldspar-carbonate matrix (Fig. 4m). Pyrochlore grains contain (wt%): 16.79–16.89 CaO, 6.71–7.55 Na_2_O, 1.23–1.44 SrO, 64.19–65.38 Nb_2_O_5_, and 1.3–3.6 F.

*Barite and synchysite-(Ce)* in pelletal lapilli consist of allotriomorphic grains and microgranular aggregates (Fig. 4m). Barite includes SrO up to 1.28 wt%. Meanwhile, synchysite-(Ce) includes up to 1.68 wt% SiO_2_, up to 0.48 wt% Al_2_O_3_, 1.38–2.89 wt% FeO_t_, 7.28–11.12 wt% CaO, with La/Ce and La/Nd ratios of 0.48–0.55, and 1.77–1.85, respectively. The presence of these phases have also been reported in the hydrothermal-metasomatic mineralization of the Chadobets damtjernites^7,14^. The crystallization of synchysite-(Ce) at the latest stages within the evolution of the UMLs hydrothermal-magmatic system also supports the evolution trend observed in fluorapatite (progressive fluorine enrichment) (Fig. 5d,e).

## Discussion

First reports of pelletal lapilli considered them as spherical to subspherical grains with a crystal or lithic core and a rim of coherent kimberlitic magma (recognizable by the microporphyritic or lath texture)^18,19,37^. Recently, some researchers include the term “pelletal lapilli” into a wide group of “magmaclasts” by introducing changes in the kimberlite terminology^38,39^. They refer to “magmaclasts” as fluidal-shaped bodies of kimberlitic magma (now solidified) formed by any process of magma disruption prior to its solidification, including melt segregations (immiscibility) and solidified melt-bearing pyroclasts^39^. These type of pyroclasts are formed by fragmentation and subsequent rapid cooling of fluidal kimberlitic magma; whereas the solidified melt segregations correspond to discrete bodies of melt formed by unmixing processes within coherent kimberlite magmas^38^.

This study adheres to the original terminology, developed and used by the following authors^18–24,40,41^, who consider pelletal lapilli as distinctive spherical formations containing in the central area a seed or kernel, which is composed of phenocrysts (macrocrysts) or lithic fragments (xenoliths), and a marginal rim (mantle) of juvenile composition of similar composition to the surrounding melt. These composite particles are formed by a fluidized flow within the explosion pipe (diatreme), which sharply crystallized the seed during the rapid rising of the melt to the surface. As noted in the introduction section, it is now thought that pelletal lapilli are originated when fluidized melts intrude into earlier volcaniclastic infill close to the diatreme root zone, meanwhile intensive degassing produces a gas jet (spray granulation), in which locally scavenged fragments (seeds) are simultaneously fluidized and coated by a spray of low-viscosity melt^25^. This model of formation can be understood as an analogue to particles formed during industrial fluidized granulation processes^42^. The example in Tappe et al.^2^ shows pelletal lapilli developed in a 1 m wide aillikite dyke, so the process may occur at the interface between the root zone and diatreme zone.

**Figure 6:**
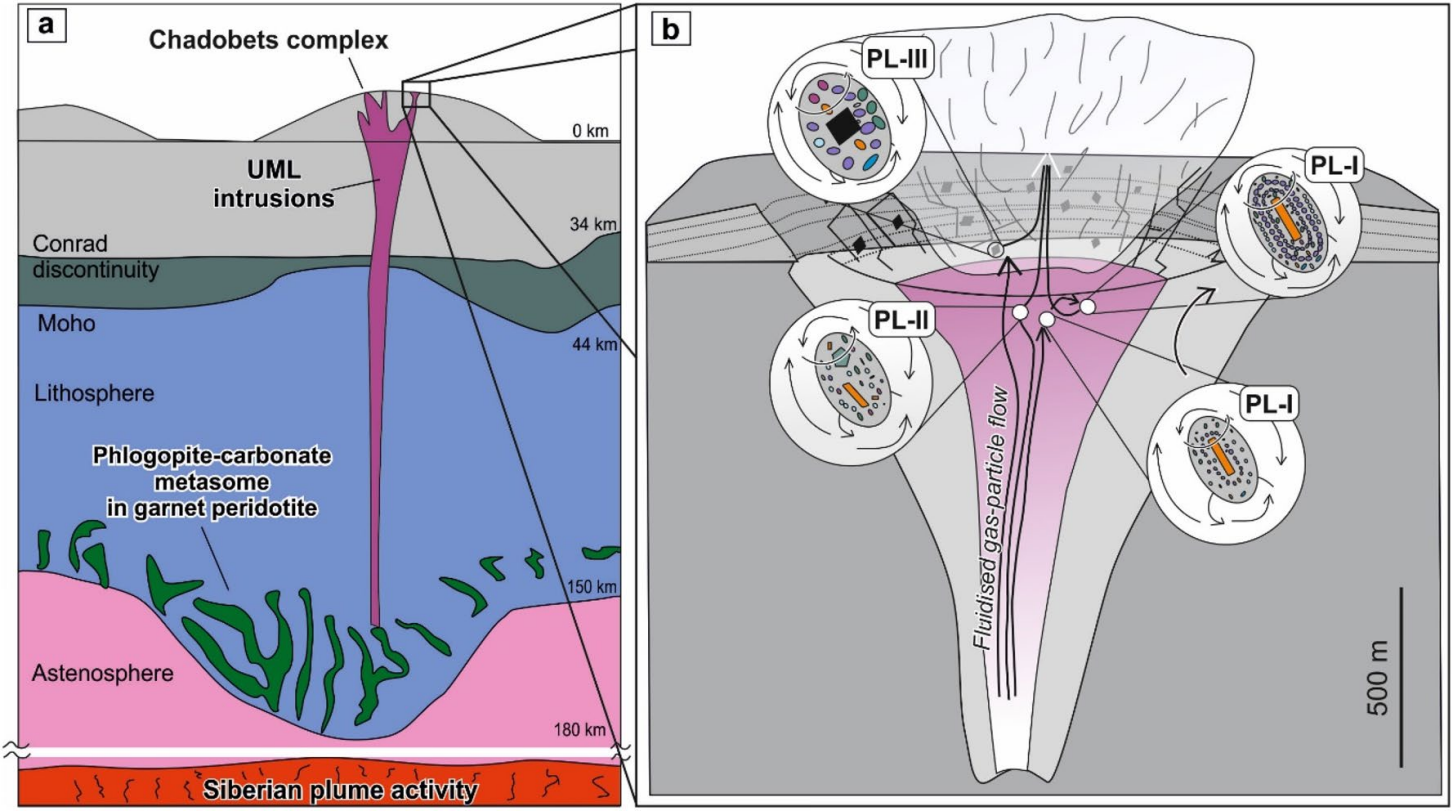
Schematic model showing the origin of the Chadobets complex UMLs from a carbonate-rich mantle source (**a**) and pelletal lapilli in damtjernites (**b**) (after^7,25^). The pelletal lapilli-I (PL-I) have a well-developed zoning, most likely formed under deep conditions in an early stage; their structure and texture evidence multiple coating of juvenile melt droplets. The pelletal lapilli-II (PL-II) presumably assembled at depths similar to the first type of pelletal lapilli or even deeper, owing to the dimension of minerals in the pelletal lapilli-II. The composition of the outer zone in the pelletal lapilli-III (PL-III), as well as the size of minerals and the composition of kernels itself (sedimentary xenoliths) indicate their near-surface origin. The figure was created using Corel Draw X4 software https://www.coreldraw.com/en/pages/coreldraw-x4/.

The mantle source region of the Chadobets complex, as determined by ɛHf-ɛNd isotopic values, was heterogeneous and characterized by the predominance of moderately depleted mantle components^7,8,10,11^. Primary melts were formed by low degree partial melting of carbonate-rich garnet peridotite (metasomatized) under the thermal influence of the Siberian plume, and did not undergo significant fractionation (e.g., Pb, Zr-Hf, and HREE)^7,8,10,11^. These early formed melts were derived from a carbonate-rich source produced by the phlogopite-carbonate metasomes within the lithospheric mantle at a depth of ~ 150–180 km^7,8,10,11^ (Fig. 6). The alkaline melts of the Chadobets complex explosively intruded the upper crust from depths of the sub-continental lithospheric mantle, whereas the latest phases of damtjernite intrusions cut through previously formed alkaline rocks to form the pelletal lapilli-bearing fluid-explosive breccia pipes (Fig. 6).

The PT-parameters of the Chadobets UMLs vary about ~ 1000–1020 °C at 20 kbar and 1200–1300 °C at 60 kbar (in olivine macrocrysts, using the Al-in-olivine thermometer)^10,14^. The data are consistent with previous temperature estimations for the Ilbokich UML complex, in olivine phenocrysts of 1240–1340 °C^43^. The calculated Δlog(*f*O_2_) FMQ values for the Chadobets UMLs are near the FMQ buffer and range from − 0.06 to − 0.22 and from − 1.02 to − 1.15 log units at 1020 °C and 1300 °C, respectively (using olivine-orthopyroxene-spinel oxygen barometer and the compositions of olivine-spinel pair)^14^.

Additionally, we applied a biotite-apatite geothermometer^44^ in order to calculate the crystallization temperature of fluorapatite and phlogopite of the Chadobets pelletal lapilli. Fluorapatite in pelletal lapilli crystallized at a late magmatic stage (see mineralogy and fluorine increasing, Fig. 5d,e) but most specifically during crystallization of relatively ferrous phlogopite, according to the well-known phlogopite evolutionary trend for the Chadobets UMLs micas^14^. Thereby, the solidus temperature estimation was estimated using the parameters of the ferrous phlogopite and the low-F fluorapatite. The results show that the original solidus temperature for the mineral pair ranges from 815 to 992 °C (Table S5). The data obtained well represent the latest stages of magmatic mineral formation of the pelletal lapilli.

Comprehensive mineralogical, structural and chemical studies of pelletal lapilli from the Chadobets complex have shown great affinity with the main mineral assemblages of damtjernites. The mineral composition of phlogopite, carbonate and fluorapatite in pelletal lapilli maps out at the start of crystallization trends for the Chadobets complex (Fig. 5), which strongly points to a juvenile composition of the mantle in pelletal lapilli from the third phase UMLs of the Chadobets intrusion. At the same time, the difference in size and composition of kernels, as well as the presence of single mineral zoning (chemical) in pelletal lapilli from damtjernites, allow us to estimate the formation conditions and depth of all three types of pelletal lapilli (Fig. 6).

Type I pelletal lapilli exhibits a distinct mineral zoning (rims), oppose to the second and third types. At the same time, the size of the mineral phases of the third type of lapilli is about an order of magnitude larger than the other two types. All these suggest different crystallization parameters influencing the genesis of pelletal lapilli. Pelletal lapilli-I, for instance, was most likely formed during the early stages of the magmatic complex evolution at deeper conditions, revealed by the complex structure and texture developed. They reflect several rising (explosive?) events within the diatreme channel and thus it is characterized by multi-trapping of juvenile melts (Fig. 6). It has also been demonstrated that the amount of vertical displacement of pelletal lapilli toward the surface, directly depends on the particle size of the constituents^45^. The larger the size, the slower the upward rate of juvenile melt droplets "melt inclusions", forming pelletal lapilli. The pelletal lapilli-II was presumably originated at the deep-middle interval of the damtjernite diatreme, at depths similar to the type I pelletal lapilli or even deeper, owing to the smaller crystal size in type II pelletal lapilli compared to pelletal lapilli-I (Fig. 6). The composition of the outer zone in type III pelletal lapilli, the size of grains, as well as the composition of the seed itself (sedimentary xenoliths) indicate their relatively near-surface genesis (Fig. 6).

Melt inclusions are small droplets of silicate melt (usually less 100 mm in size) that are trapped in minerals during their growth in magma^46^. This melt can be trapped within a host mineral by various mechanisms. One of them is through surface irregularities that create crystal defects, comparable to when an adhering mineral grain or vapor bubble leads to the formation of an embayment that is eventually engulfed due to subsequent crystal growth^46^. Another common mechanism consists of melt trapping in response to periods of rapid crystal growth followed by slower growth, leading to the enclosing of numerous small inclusions along growth zones. In our investigation, we observed clear evidence of a slightly different mechanism capable of enclosing and preserving small portions (droplets) of fluidized melt, through a rapid crystallization of the melt blebs on the flowing pelletal lapilli seeds. All the above arguments allow us to propose that pelletal lapilli should be considered as a type of melt inclusion existing in ultramafic lamprophyres and related rocks such as kimberlites and lamproites.

## Methods

For the whole investigation, core samples of damtjernites from exploratory wells of the Chuktukon REE-Nb deposit in the Chadobets UML-carbonatite complex were used. Analytical methods and techniques such as X-ray microspectral (microprobe) analyzes, scanning electron microscopy (SEM) and Raman structural analyzes, including areal mapping with a 2 μm step to diagnose the volumetric (3D) ratio of mineral phases in aggregates of the pelletal lapilli; were performed for a comprehensive study of the samples.

During the petrographic investigation phase, polished samples of both thin-sections and resin mounted (for ore-mineralogy) were prepared and studied in transmitted and reflected light, respectively, using a petrographic microscope Olympus BX51 coupled with a HD camera. Additionally, the epoxy mounted-samples helped in determining the rock textures and mineral assemblages using energy-dispersive spectrometry in combination with back-scattered electron imaging (BSE) in a TESCAN MIRA 3 LMU JSM-6510LV scanning electron microscope with energy-prefix X-Max by Oxford Instruments. Mineral compositions were determined using an electron microprobe JEOL JXA-8100 (WDS mode, 20 kV, 15 nA, 1–2 μm beam diameter). Accumulation time for analyzing of F (using LDE crystal) was 40 s (20 s—counting of background; 20 s—counting of peak for F), detection limit for F was 477 ppm (0.04 wt%). For every mineral analysis run, we used a beam current of 10 nA and an acceleration voltage of 15 kV; for Fe-Ti oxides 20 nA and 15 kV, for monazite 40 nA and 20 kV, for apatite 10 nA and 20 kV. The peak counting time was 16 s for major elements and 30–60 s for minor elements. Calibration was carried out using as standards both, natural minerals and synthetic phases (element, detection limits in ppm): SiO_2_ (Si, 158), rutile (Ti, 120), LiNbO_3_ (Nb, 142), Sr silicate glass (Sr, 442), albite (Na, 176), orthoclase (K, 182), Al_2_O_3_ (Al, 128), F-apatite (Ca, 115; P, 387; F, 477), Mn-garnet (Mn, 129), hematite (Fe, 148), CePO_4_ (Ce, 236), LaPO_4_ (La, 272), BaSO_4_ (S, 178), NdPO_4_ (Nd, 362), Cl-apatite (Cl, 74), and PrPO_4_ (Pr, 401).

Raman spectroscopy was performed to determine the composition of mineral phases in PL using a LabRam HR800 Horiba Jobin Yvon spectrometer, equipped with an optical microscope (Olympus BX41). The 514.5 nm Ar^+^ laser line was used for spectra excitation. Well known RRUFF (http://rruff.info) database for Raman spectra was used in the identification of solid phases. In addition, the mineral-structure compositional study of pellet lapilli, 2D mapping was carried out in several samples in depth using a Raman spectrometer with automatic confocal Raman imaging WITec Apyron. A 488 nm laser (50 mW) was applied to excite the sample. The phase separation and mapping were performed using the “True component analysis” algorithm of the WITec Project FIVE + software. The baseline, cosmic peaks, and partially the lines of neighboring phases were subtracted from the spectra. All analytical methods were carried out at the Analytical Center for multi-elemental and isotope research Siberian Branch Russian Academy of Science (Novosibirsk, Russia).

## Supplementary Information

Supplementary Legends.

Supplementary Table S1.

Supplementary Table S2.

Supplementary Information 4.

Supplementary Table S5.

## Data Availability

The original data generated and analyzed during this study are included in this article and its supplementary information files. Additional datasets used during the current study are also available from the corresponding author on reasonable request.
