# Supplementary material for: Petrogenesis of juvenile pelletal lapilli in ultramafic lamprophyres
Source: Sci Rep. 2023 Apr 10;13:5841. doi: 10.1038/s41598-023-32535-2 (PMC10086001; doi:10.1038/s41598-023-32535-2)
Supplement: Supplementary file 1 — Supplementary Legends. [file 41598_2023_32535_MOESM1_ESM.docx]

**CAPTIONS OF THE SUPPLEMENTARY MATERIALS**

**Supplementary Table 1S.** Chemical composition of phlogopite (EPMA data, wt.%).

**Supplementary Table 2S.** Chemical composition of carbonate (EPMA data, wt.%).

**Supplementary Table 3-4S.** Chemical composition of fluorapatite (EPMA data, wt.%).

**Supplementary Table 5S.** Measurements of the crystallization temperature of fluorapatite and phlogopite of the Chadobets pelletal lapilli according to biotite-apatite geothermometer.
